# Dynamic monitoring of myeloma minimal residual disease with targeted mass spectrometry

**DOI:** 10.1038/s41408-023-00803-z

**Published:** 2023-02-24

**Authors:** Somayya Noori, Charissa Wijnands, Pieter Langerhorst, Vincent Bonifay, Christoph Stingl, Cyrille Touzeau, Jill Corre, Aurore Perrot, Philippe Moreau, Hélène Caillon, Theo M. Luider, Thomas Dejoie, Joannes F. M. Jacobs, Martijn M. van Duijn

**Affiliations:** 1 Department of Neurology, Erasmus University Medical Center, Rotterdam, the Netherlands; 2 Department of Laboratory Medicine, Radboud University Medical Center, Nijmegen, the Netherlands; 3 Sebia, Lisses, France; 4 Centre Hospitalier Universitaire de Nantes, Nantes, France; 5 Unite de Genomique du Myelome, Institut Universitaire du Cancer de Toulouse-Oncopole, Toulouse, France; 6 Institut Universitaire du Cancer de Toulouse-Oncopole, Toulouse, France; 7 Hematology, University Hospital Hôtel-Dieu, Nantes, France; 8 Biochemistry Laboratory, Hospital of Nantes, Nantes, France

**Keywords:** Myeloma, Immunology

Dear Editor,

Minimal residual disease (MRD) assessment has been established as a prognostic indicator for multiple myeloma (MM) [1]. MRD-negativity correlates with longer progression-free survival and has the potential to become a surrogate endpoint in future clinical trials [2, 3]. Current methods used in clinical trials for MRD detection, such as next-generation sequencing (NGS) and flow cytometry, are sensitive enough to detect one myeloma cell in ≥ 10^5^ cells in bone marrow [4]. However, the invasive character of bone marrow aspiration and the heterogeneous distribution of myeloma cells limits the application [5]. For monitoring the monoclonal protein (M-protein) in blood, methods such as serum protein electrophoresis (SPEP) and free light chain (FLC) analysis remain the gold standard. However, these electrophoretic methods are not sensitive enough to detect low disease burden in patients with stringent complete remission.

Blood-based targeted mass spectrometry (MS) assays have shown to be at least 1000 times more sensitive than conventional electrophoretic methods [6–8]. In a retrospective study, we recently showed a concordance of 79% between MRD status in blood by MS (MS-MRD) and in bone marrow by NGS (NGS-MRD), and similar prognostic value of both methods in a cohort of 41 newly diagnosed MM patients enrolled in the Intergroupe Francophone du Myélome (IFM) 2009 study [9, 10]. The sensitivity of MS-MRD has the potential to enable quantitation of disease activity in MM patients with stringent complete remission, while the minimally invasive character of MS-MRD also allows for more frequent sampling. In this study, we investigated the feasibility of blood-based dynamic MS-MRD monitoring in 926 longitudinal serum samples from the same 41 patients studied in the previous MS-MRD study [9].

From the IFM 2009 clinical trials (ClinicalTrials.gov identifier NCT01191060), 41 MM patients were selected based on the availability of RNA-sequencing data, NGS-MRD data, and follow-up serum samples. In the previous MS-MRD study [9], we assessed three serum samples of each patient and compared our data to NGS-MRD. In this study, we have assessed all 926 available serum samples, which amounted to 13 to 31 follow-up samples per selected patient for dynamic MS-MRD monitoring (Supplementary Table 1). Serum samples were digested with trypsin and analyzed with targeted parallel reaction monitoring mass spectrometer. Data were processed with Skyline analysis software, whereby the software tool Peakfit was used to automatically select the correct peptide peak boundaries before data was manually reviewed [11]. M-protein serum levels were quantified in sera of all 41 patients based on patient-specific clonotypic M-protein peptides (Supplementary Table 2). These clonotypic peptides were selected from the M-protein sequences obtained through bioinformatics analysis from RNA-sequencing data obtained in the IFM trial, as described previously [9]. MS-MRD progression was determined based on the slope of log-transformed M-protein concentration. A detailed description of the methods is explained in the Supplementary Methods.

MS-MRD is able to reproduce the M-protein trends in response kinetics determined with SPEP and additionally improves the results through higher sensitivity. The deepest response detected with MS-MRD was at 0.3 mg/L M-protein. MS-MRD could monitor the M-protein in 864 of the 926 serum samples, SPEP detected the M-protein in only 195 of the serum samples, and FLC measured the M-protein in 230 of the serum samples with an abnormal FLC kappa-to-lambda ratio (Supplementary Fig. 1), four of these examples are shown in Supplementary Fig. 2. MS-MRD was also compared to NGS-MRD, whereby a concordance of 78% (Supplementary Table 3) was achieved, which is similar to the concordance achieved previously [9]. Considering that NGS-MRD detects clonal DNA in bone marrow and that MS-MRD detects the M-protein in blood, some discrepancies in MRD assessment can be expected. Cases where MS-MRD shows disease positivity whereas NGS-MRD is negative for the same patient could have been caused by sampling bias. Extramedullary myeloma, the patchy nature of MM in bone marrow, or hemodilution in the bone marrow sample could all be the cause of NGS-MRD negativity. In these instances, dynamic MS-MRD monitoring gives additional information about disease progression.

Figure 1 shows four examples of the visualization of the dynamic MS-MRD monitoring in all 41 patients. In Fig. 1A, the patient’s M-protein was monitored in all 18 serum samples collected over 909 days. During the induction phase, a decrease of M-protein is accompanied by loss of signal with SPEP but not with MS-MRD. We assessed signs of progression in the patient by quantifying rates of change; already during maintenance therapy our criteria were met, and MS-MRD progression was declared. The M-protein was detectable again with SPEP one year later, whereupon the progression criteria of the trial were met as well. This example shows that MS-MRD observed different M-protein profiles after treatment between the 41 patients. Trends in M-protein levels frequently changed following changes in the treatment regimen, which highlights the importance of dynamic MRD monitoring. For six patients, M-protein levels already started rising during the maintenance period, and in 14 patients rising levels were observed within one year of the end of maintenance treatment. Another nine patients showed such a rise more than one year after the end of maintenance treatment, whereas the remaining 12 patients did not show evidence of an increase throughout the entire period of disease follow-up, or M-protein signals were lost entirely. Figure 1A–D shows examples of such disease profiles; all patients’ profiles are available in Supplemental Figs. 3–6. The monitoring profiles of three patients were reproduced in three independent experiments (Supplementary Fig. 7) and were also repeated in a different laboratory and instrument platform, yielding the same data in the cross-validation. (Supplementary Fig. 8).

**Fig. 1:**
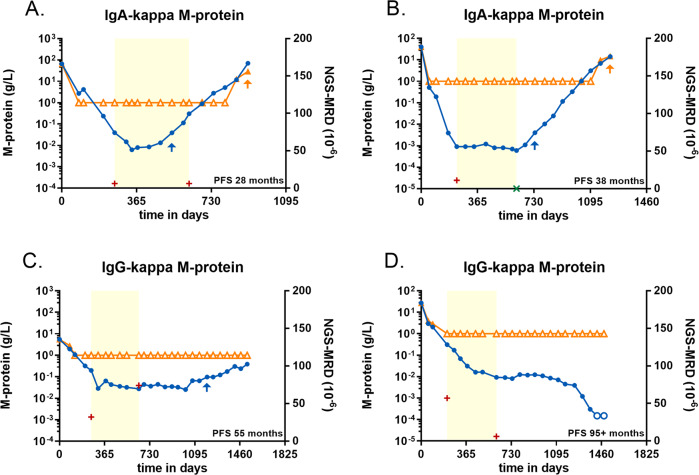
Dynamic monitoring profiles in the IFM 2009 cohort. Open symbols indicate the analyte could not be detected. Orange triangles show SPEP data for the M-protein; blue dots show MS-MRD data. The yellow area indicates the period of maintenance treatment, with NGS-MRD assessments at the start and end. A positive NGS-MRD result is shown as a red plus; a negative NGS-MRD result as a green X. The blue arrow shows early evidence of progression based on MS-MRD data; the orange arrow shows where progression was seen in the data from the IFM 2009 study. **A** Patients with early progression during maintenance treatment. Patient 020001, IgA-kappa M-protein. **B** Patients with progression within one year after end of maintenance treatment. Patient 052003, IgA-kappa M-protein. **C** Patients with progression more than one year after end of maintenance treatment. Patient 044012, IgG-kappa M-protein. **D** Patients with no progression observed, this particular patient reached MS-MRD negativity after 1400 days of follow-up. Patient 025007, IgG-kappa M-protein.

The patient cohort that was measured included patients from both treatment arms of the IFM 2009 study. These arms (A and B) mainly differed by the use of RVD (lenalidomide, bortezomib, and dexamethasone) with or without intensive treatment by melphalan plus autologous stem cell transplantation. Based on the M-protein profiles obtained with MS-MRD, we observed increases in M-protein during the maintenance treatment for a subset of patients in arm A, whereas all transplanted patients in arm B showed a persistent decrease in M-protein throughout the maintenance treatment (Supplementary Fig. 9). There is also a difference in the rate of M-protein clearance dependent of the M-protein isotype. Supplementary Fig. 10 shows examples of therapy response-kinetics for each M-protein isotype found in the cohort, which reflect the expected half-life of Ig isotypes (IgA, 6 days; IgG, 23 days; FLC, 6 h). The expected half-life defines an upper limit to the expected clearance of M-protein—even in the case of a complete and immediate response to treatment [12, 13]. An M-protein decrease close to the typical Ig half-life for its isotype could indicate an optimal treatment response, whilst a slower clearance could indicate a still considerable production of M-protein by a remaining population of myeloma cells, sufficient to offset the clearance. Additionally, in some patients we found an increase of serum levels of light chain clonotypic peptides compared to heavy chain clonotypic peptides, which might represent early detection of light chain escape (Supplementary Fig. 11).

According to the trial criteria, 13 of the 41 patients progressed from complete response during the serum sample collection period. When defined by MS-MRD data, signs of progression were seen earlier in 12 out of these 13 patients and never later. On average, progression was detected 442 days earlier with MS-MRD than in the original trial (*p* < 0.0001) (Supplementary Fig. 12). One of the 28 other patients progressed within the serum sample collection period while never reaching complete response, whereas 21 patients progressed after the serum collection period and six did not. In 15 of these 21 patients, we did observe early signs of progression by MS-MRD in the collected serum samples. However, one of those did not progress by other criteria (patient 067005: 714 days), Supplementary Fig. 4M. Currently, early detection of disease progression does not have an impact on managing relapse, and it is unclear if a longer continuation of maintenance therapy could have prevented such progression. However, MS-MRD provides the opportunity to evaluate such questions in clinical trials and spark interest in both the understanding of MM evolution in patients and the perspective of adapting treatment strategy to the patient’s response.

In conclusion, MS-MRD was found to be feasible on a retrospective cohort of 41 MM patients, it has shown reproducibility of the protocol on two different mass spectrometers in two different laboratories, and it has provided good concordance with bone marrow-based NGS-MRD. Additional clinical validation is needed to assess the value of MS-MRD in clinical trials. Dynamic MS-MRD monitoring in retrospective and prospective patient cohorts will enable defining progression in a reliable way, and has likely complementary value in MM patients with extramedullary disease. MS-MRD could aid to optimize the timing of bone marrow-based evaluation and direct the need for additional or confirmatory bone marrow aspirates. Dynamic MS-MRD monitoring opens ways to treat patients more individually and to optimize the effects of treatment possibilities for MM patients.

## Supplementary information

Supplemental Information

## Data Availability

MS data and Skyline files are available in the public data repository Panorama Public (https://panoramaweb.org/MSMRDmonitoring.url).
