# Supplementary material for: Dynamic monitoring of myeloma minimal residual disease with targeted mass spectrometry
Source: Blood Cancer J. 2023 Feb 24;13(1):30. doi: 10.1038/s41408-023-00803-z (PMC9957984; doi:10.1038/s41408-023-00803-z)
Supplement: Supplementary file 1 — Supplemental Information [file 41408_2023_803_MOESM1_ESM.docx]

Supplemental Information

**Supplementary Materials and Methods**

**Reagents:** A total of 79 synthetic stable isotope-labelled peptides were purchased from Pepscan (Lelystad, the Netherlands). Ammonium bicarbonate and dithiotreitol were purchased from Sigma-Aldrich (Saint Luois, MO, USA). Rapigest surfactant was purchased in 10 mg lyophilized vials from Waters (Milford, MA, USA). Iodoacetamide was purchased in single use vials of 56 mg from Sigma-Aldrich. Trypsin was purchased in 100 µL lyophilized vials from Millipore (Darmstadt, Germany). Acetonitrile, trifluoroacetic acid, formic acid, and LC-MS water were purchased from Biosolve (Valkenswaard, The Netherlands).

**Patient serum samples:** From 2010 to 2012, a phase three IFM clinical trial enrolled 700 patients under the age of 66 years who were eligible for autologous stem cell transplantation (ASCT). The patient cohort was divided into two treatment arms: patients in arm A received three cycles of RVD (lenalidomide, bortezomib, and dexamethasone), followed by additional five cycles of RVD. Patients in arm B also received three cycles of RVD, but next received melphalan and underwent ASCT, then were given two additional cycles of RVD. In this study, we have assessed all 926 serum samples of 41 out of the 700 patients. These were 13 to 31 serum samples per patient collected longitudinally over 29 to 60 months. **Supplementary Table 1** provides the total number of serum samples per patient and relevant patient information.

**Qualified clonotypic peptides:** In the previous study, bioinformatics analysis was performed on available RNA-sequencing data from the IFM trial to obtain the M-protein sequences of all 41 patients. Clonotypic peptides of these M-protein sequences were selected for detection. The diagnostic serum samples were prepared for MS analysis according to our standard digestion protocol, described in the previous study. In silico predicted tryptic peptides of the M-protein sequence that had mutations compared to the immunoglobulin germline reference were evaluated with targeted MS. Additionally, clonotypic peptides matching the variable domain of the M-protein were evaluated for their uniqueness in the target patient in comparison to the other patient samples, which is commonly used criteria for clonotypic peptide candidates in SRM/MRM assays. Experimentally validated clonotypic peptides for each patient were quantified in serum with synthetic stable isotope-labelled peptides (Pepscan, Lelystad, the Netherlands). In this study, the same qualified clonotypic peptides and their stable isotope-labelled peptides as previously described were used for MS-MRD monitoring (**Supplementary Table 2**).

**Sample preparation:** In a 96 well plate (Axygen, Corning, NY, USA), 2 µL of patient serum samples were diluted into 500 µL 50 mM ammonium bicarbonate with 27 % acetonitrile. Serum sample dilutions were treated with 30 µL 0.2 % Rapigest and 100 ng of a heavy-isotope labelled intact immunoglobulin was added for later digestion quality control. Samples were reduced with 10 mmol/L dithiotreitol for 30 min at 60 °C and 300 rpm in a sealed plate. Samples were cooled at 4 °C for 10 min and centrifuged (3 min, 4000 rpm). Samples were alkylated with 14 mmol/L iodoacetamide for 30 min at room temperature. Overnight digestion was performed with 4 µL 100 ng/µL trypsin (Promega, Madison, WI, USA). After cooling (10 min, 4 °C) and centrifuging (3 min, 4000 rpm), samples were acidified with 1.5 µL 25% trifluoroacetic acid and incubated 30 min at 37 °C and 300 rpm. Samples were cooled again and filtered over a pre-wetted 0.45 µm Multiscreen-HA filter plate (Millipore, Merck, Darmstadt, Germany). Then, 2 µL of the resulting digestion was loaded in the mass spectrometer for analysis.

**Mass spectrometry measurements:** A 30 min peptide separation in 2 to 31 % acetonitrile in 0.1 % aqueous formic acid was performed on an EasySpray C18 column (0.15 mm x 100 mm) and measured with targeted MS (Orbitrap Exploris 120, Thermo Fisher Scientific, Waltham, MA, USA). The following MS parameters were applied: 60000 resolution, 2 m/z isolation window, an internal mass calibration with a lock mass of 445.12003 m/z, after every 20 spectra an MS1 scan was included, RF lens of 70%. Each clonotypic peptide was measured with previously optimized collision energy between 18 to 36 normalized collision energy (**Supplementary Table 2**). For controls, synthetic peptide mixtures were injected before and after the patient measurements to validate LC-MS performance. For each patient, serum samples that were negative by SPEP were measured first in order to limit any influence of carry-over effects. A triplicate mixture of the serum samples taken at entry of each patient was measured with data dependent acquisition to validate digestion performance for each experiment. All serum samples of 3 patients were experimentally prepared and measured in 3 independent experiments over a period of 6 months. Additionally, the 3 patients were also experimentally prepared in a second laboratory and measured on a different LC and MS system (timsTOF Pro, Bruker, Leiderdorp, The Netherlands). In order to characterize the reproducibility of the sample preparation and analysis that was used.

**Data analysis:** Skyline version 21.2 was used to quantify the patient M-protein concentration (g/L) based on the clonotypic peptides measured in each serum sample with the targeted MS. Additionally, the dot product between endogenous and reference peptide signals detected in the mass spectra was used to accept or reject peptides as valid signals (threshold 0.89). The dot product is calculated by Skyline based on the ratio between the light transition peak areas and heavy transition peak areas of the targeted clonotypic peptide. Based on this threshold, one of the two quantified proteotypic peptides of the patient’s M-protein was selected to qualify for the M-protein, for patients with FLC or light chain escape M-protein analysis was made on the light chain clonotypic peptide. For all patients who were SPEP positive at diagnosis the M-protein concentration quantified with MS-MRD was adjusted to the M-protein concentration of the SPEP data at diagnosis. MS-MRD results were compared to not only available SPEP data, but also to the kappa-to-lambda ratio’s from the FLC measurements with reference intervals of 0.26 to 1.65. These FLC measurements were performed on a BNII nephelometer (Siemens Healthcare) with Freelite reagents from The Bindings Site, all according to the manufacturer’s protocol. For assessing progression in MS-MRD data, linear regressions were performed on four time points of log-transformed data. At the first occurrence of a slope and r^2^ exceeding thresholds (5·10-3 ln[g/L]/day; 0.8) a progression was declared. Graph Pad prism version 9.3.1 was used to prepare figures. Peaks studio version 10.5 was used to evaluate the digestion in quality controls.

**Supplementary Figures and Figure Legends**


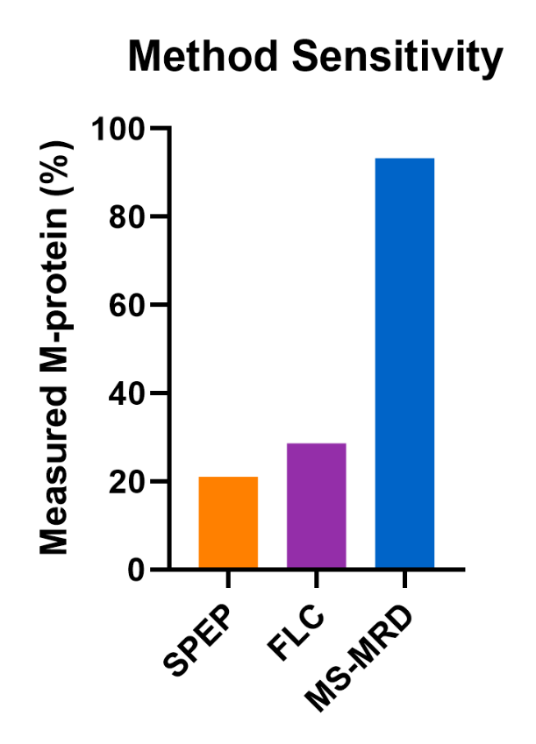


**Supplementary Figure 1: Comparison of method sensitivity between SPEP, FLC, and MS-MRD.** A total of 926 serum samples were measured with SPEP (orange), FLC (purple), and MS-MRD (blue). Out of the 926 samples, M-protein was detected in 195 (21.06%) samples by SPEP, in 230 (24.8%) samples by FLC measurements, and in 864 (93%) samples by MS-MRD.

**
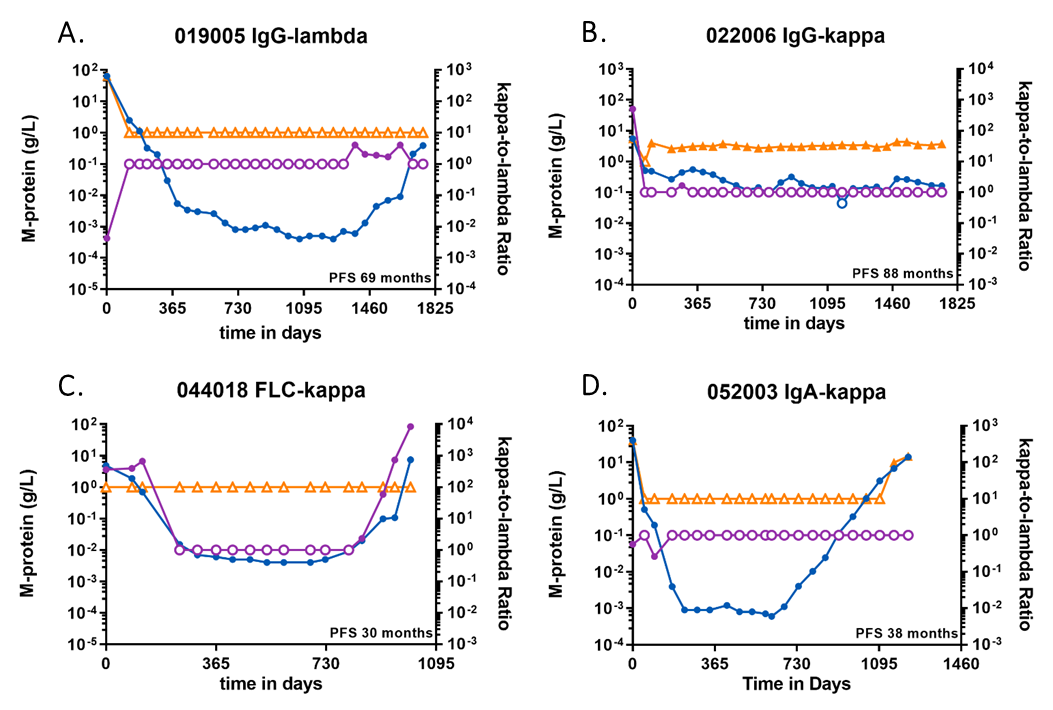
**

**Supplementary Figure 2: Comparison between MS-MRD and FLC in 4 patients.** The ratio between the kappa and lambda determined by FLC (purple) is shown on the right y-axis. Abnormal FLC ratio is visualized with closed symbols and normal FLC ratio is shown with open symbols. Patient 019005 has lambda light chain M-protein. Patient 022006, 044018, and 052003 have kappa light chain M-protein. Patient 044018 has only FLC M-protein. Based on MS-MRD (blue), M-protein increase is seen in patient 019005, 044018, and 052003 within the collected time points, while patient 022006 does not progress. Based on FLC, progression is detected only in patient 044018 and the other 3 patients did not show progression. In the cohort of 41 MM patients, MS-MRD detected in most cases an increase of M-protein while FLC did not detect progression or detected progression later than MS-MRD. The M-protein detected by SPEP (orange) is shown with closed symbols and not detected M-protein is shown with open symbols.

**
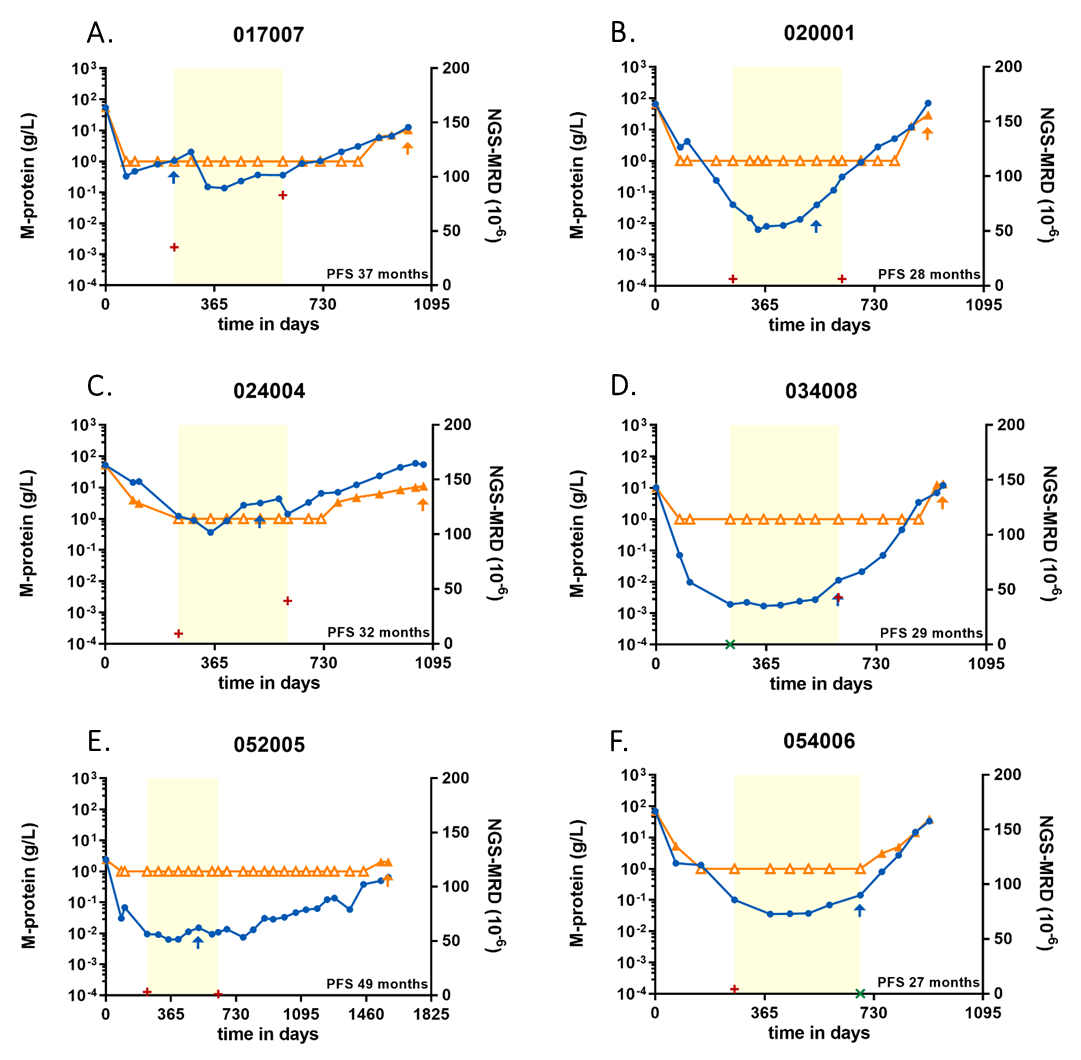
**

**Supplementary Figure 3: M-protein increase during maintenance treatment seen in 6 patients from the cohort.** Open symbols indicate the analyte could not be detected. Orange triangles show SPEP data for the M-protein; blue dots show MS-MRD data. The yellow area indicates the period of maintenance treatment, with NGS-MRD assessments at the start and end. A positive NGS-MRD result is shown as a red plus; a negative NGS-MRD result as a green X. The blue arrow shows early evidence of progression based on MS-MRD data; the orange arrow shows where progression was seen in the data from the IFM 2009 study. Majority of these 6 patients show a rapid M-protein increase and have therefore a poor PFS. Which brings the average PFS for these 5 patients to only 34 ± 8 months.

**
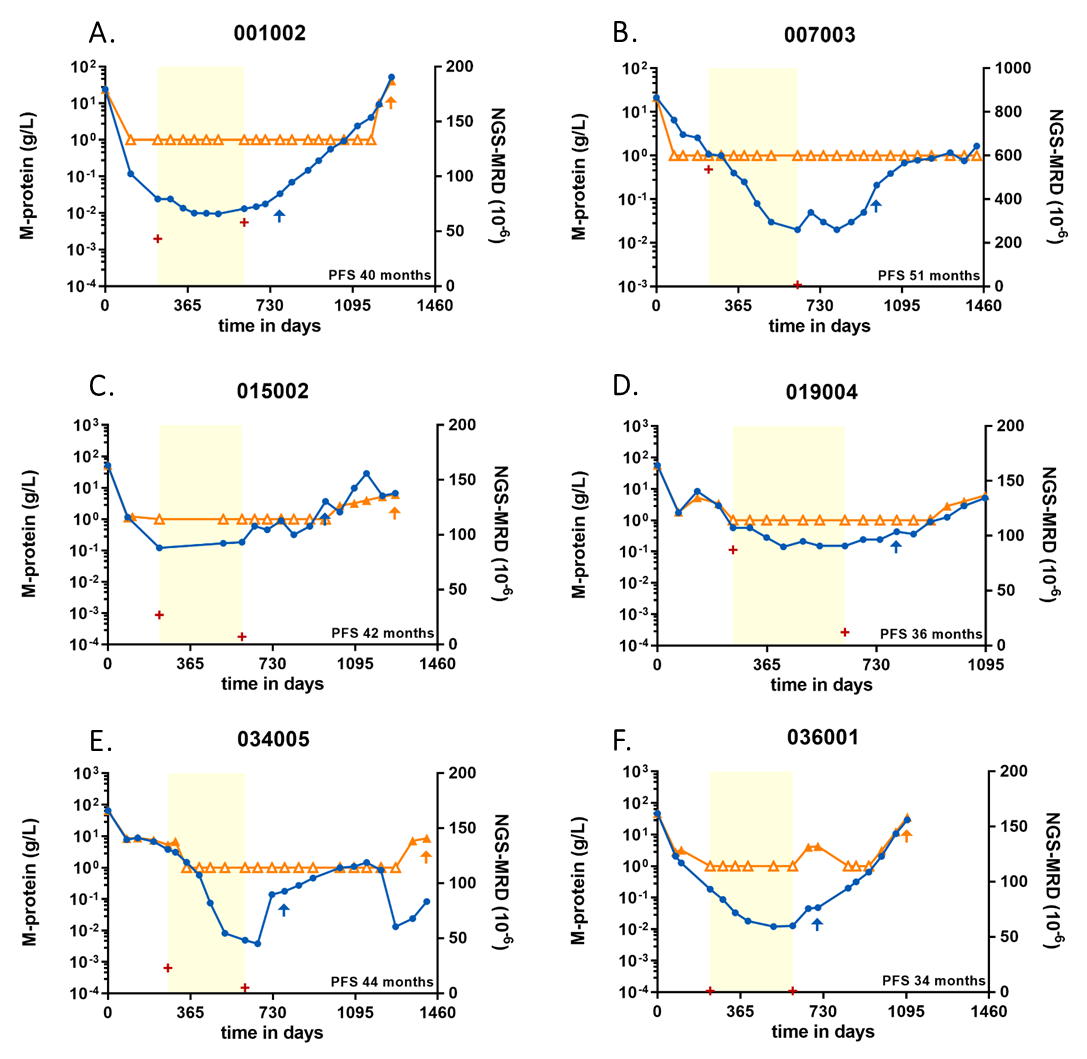
**

**
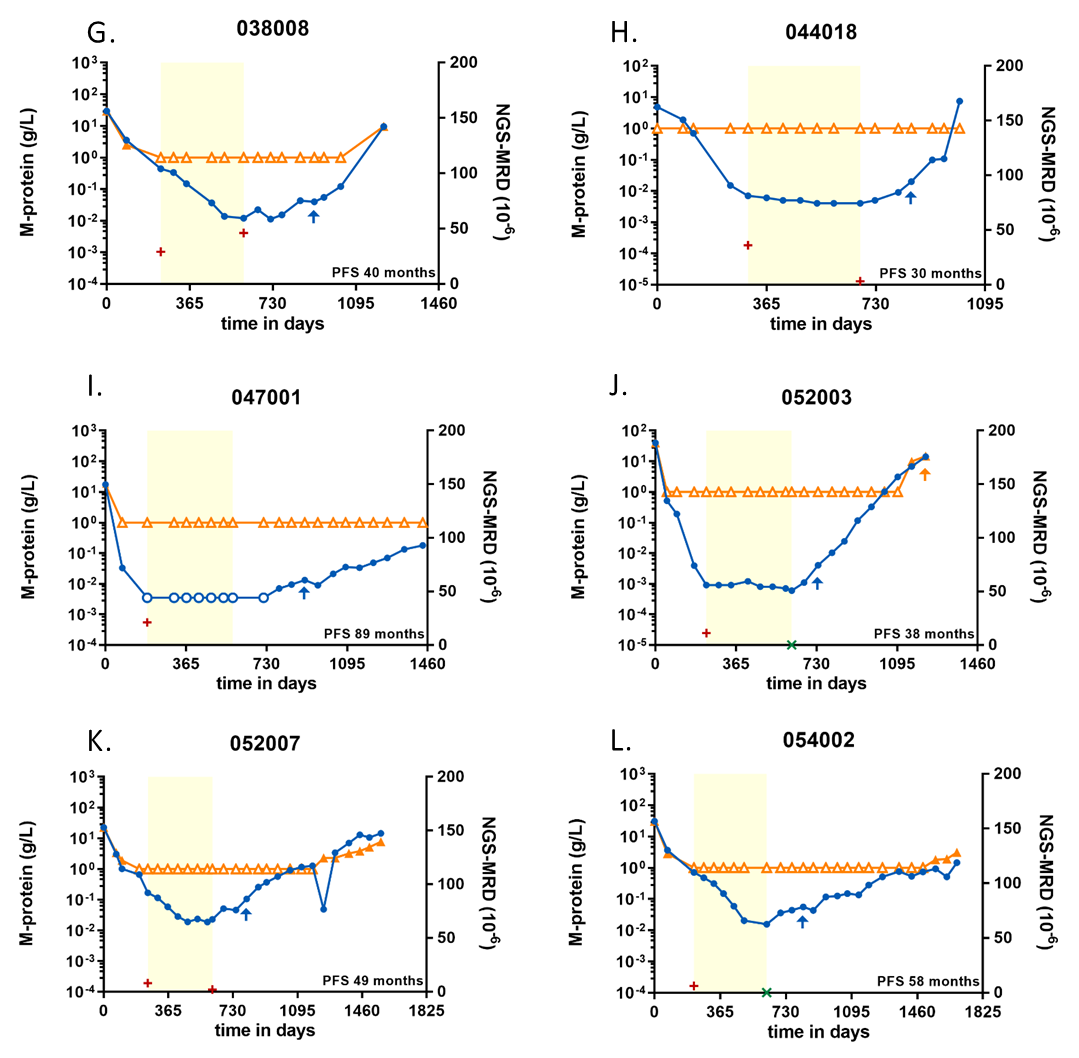
**

**
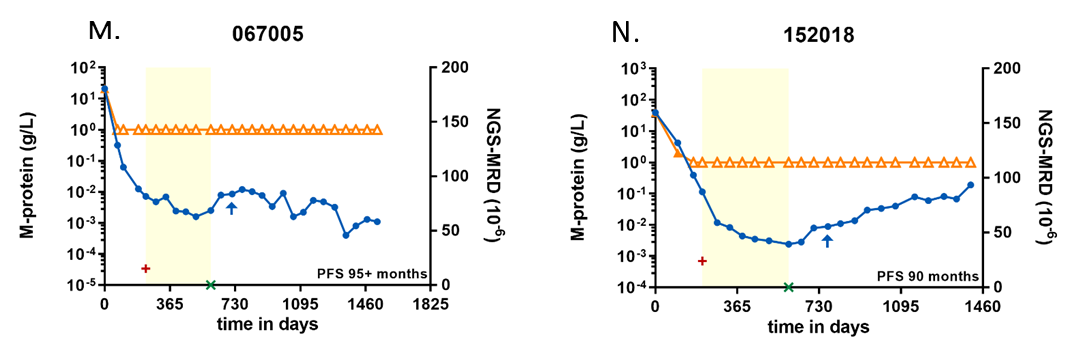
**

**Supplementary Figure 4: M-protein increase within 1 year after the end of maintenance treatment seen in 14 patients of the cohort.** Majority of these 14 patients have a poor PFS, with one exception who did not relapse (067005) in the IFM2009 clinical trial. For these 19 patients, the average PFS is just 53 ± 22 months.

**
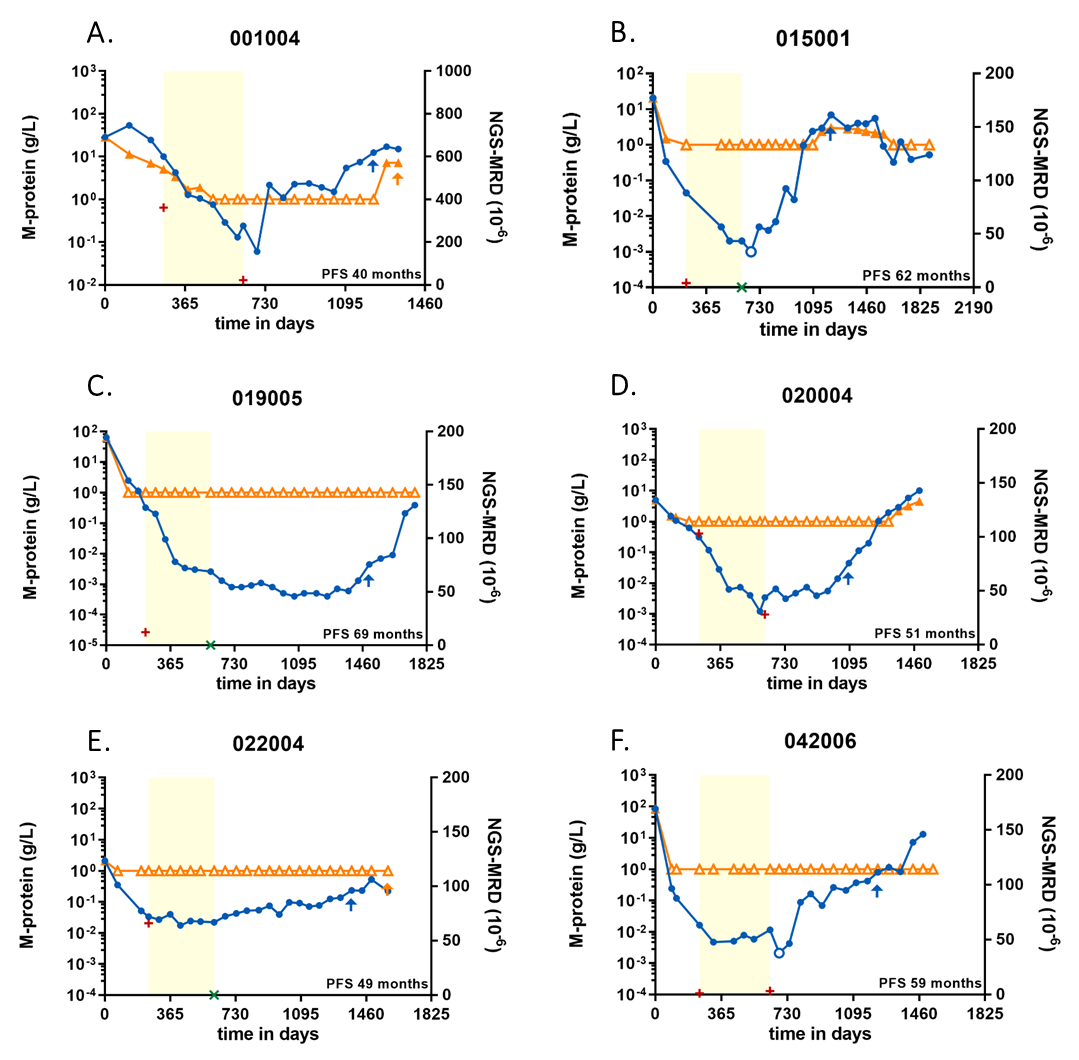
**

**
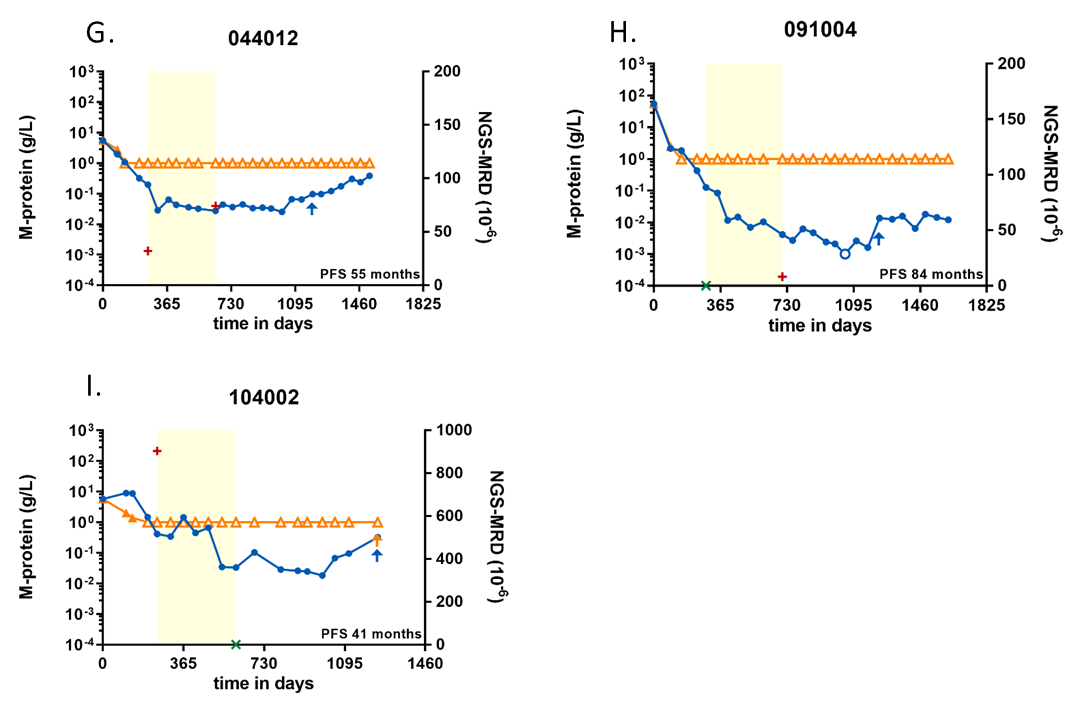
**

**Supplementary Figure 5: M-protein increase more than 1 year after the end of the maintenance treatment seen in 9 patients of the cohort.** Some patients increase after a period of deep response (019005, 020004), other patients show increase at the last time point of the measured sampling period. The average PFS is 57 ± 14 months.

**
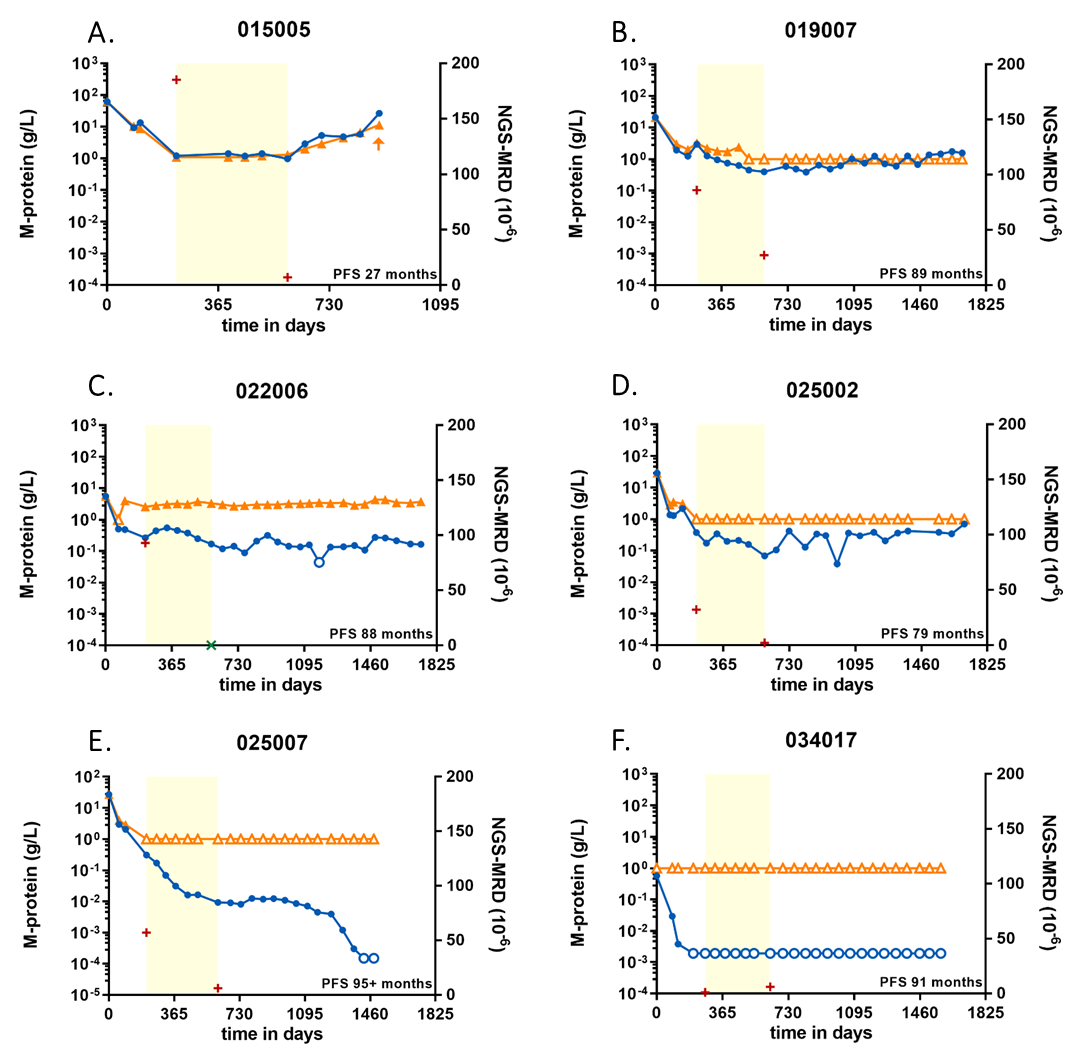
**

**
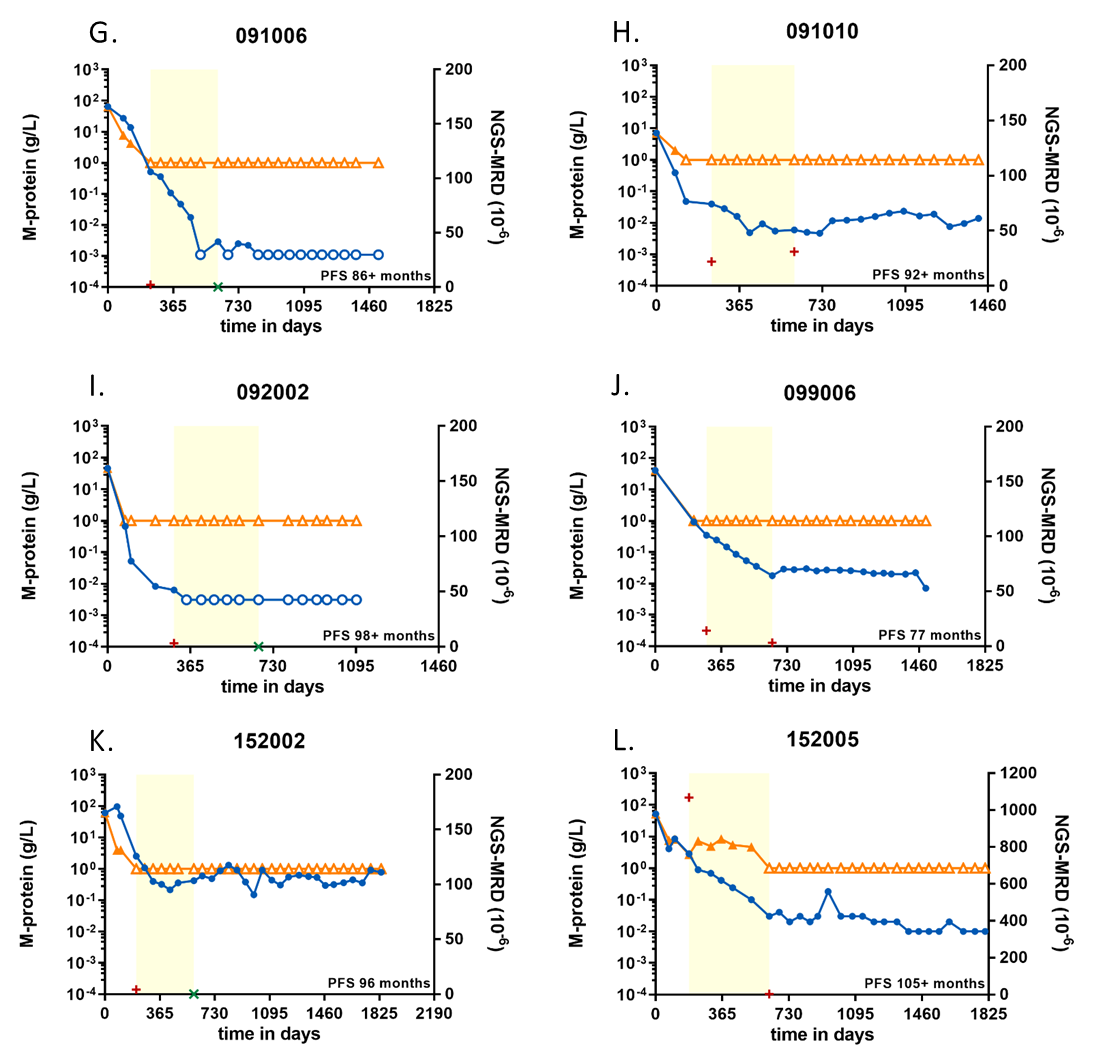
**

**Supplementary Figure 6: M-protein increase not observed in 12 patients of the cohort.** The M-protein in these patients decrease and 4 of the patients become MS-MRD negative by the end of the course of disease. These patients have the longest PFS on average, which is 85 ± 20 months.


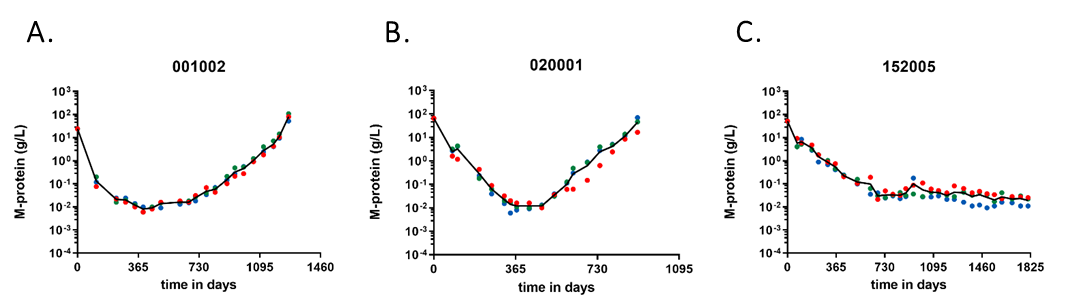


**Supplementary Figure 7: Assay reproducibility.** The results of 3 patients are shown for samples that have been prepared and measured at 3 independent times (blue, green, and red markers). The average of all 3 digests is shown with a black line. The average CV is 28% (001002), 43% (020001), and 43% (152005).

**
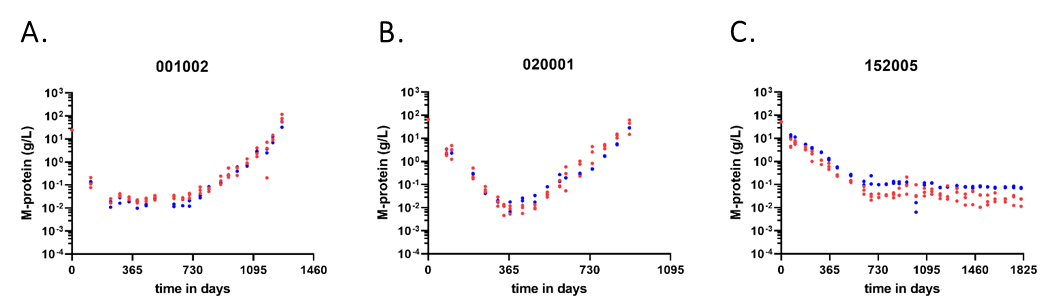
**

**Supplementary Figure 8: Assay portability.** The results of 3 patients are shown for samples that have been prepared and measured at two independent labs with different LC-MS equipment. The data in red was obtained with Orbitrap MS as all other data in this study and the data in blue was obtained on a TIMS-TOF (Bruker, Germany). The overall correlation coefficient is 0.97 between the data of both laboratories.

**
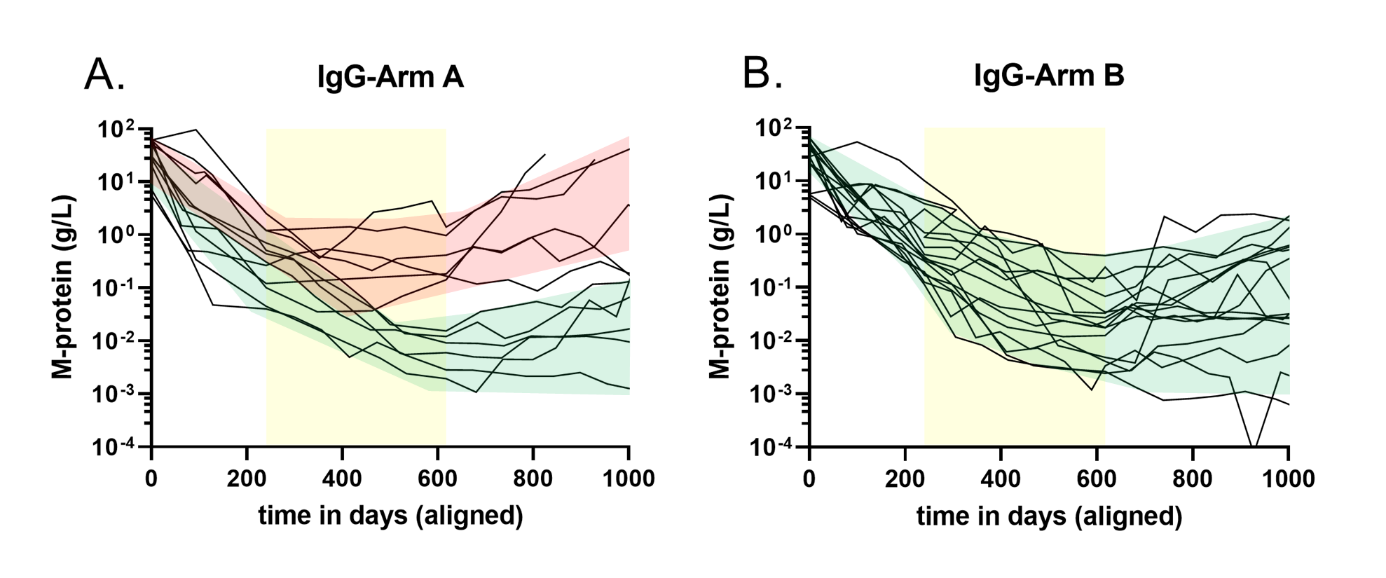
**

**Supplementary Figure 9.** **Distinct profiles for treatment arms.** A) Shown are dynamic MS-MRD profiles for all patients with an IgG M-protein in Arm A of the IFM 2009 trial (n=12). B) Dynamic MS-MRD profiles for all patients with an IgG M-protein in Arm B of the IFM 2009 trial (n=16), which included ASCT. There is an increase of M-protein in a subset of patients from Arm A (red) compared to the rest of the patients in both treatment arms (green). All profiles were scaled in the time domain to align the start and ending of the maintenance treatment.

**
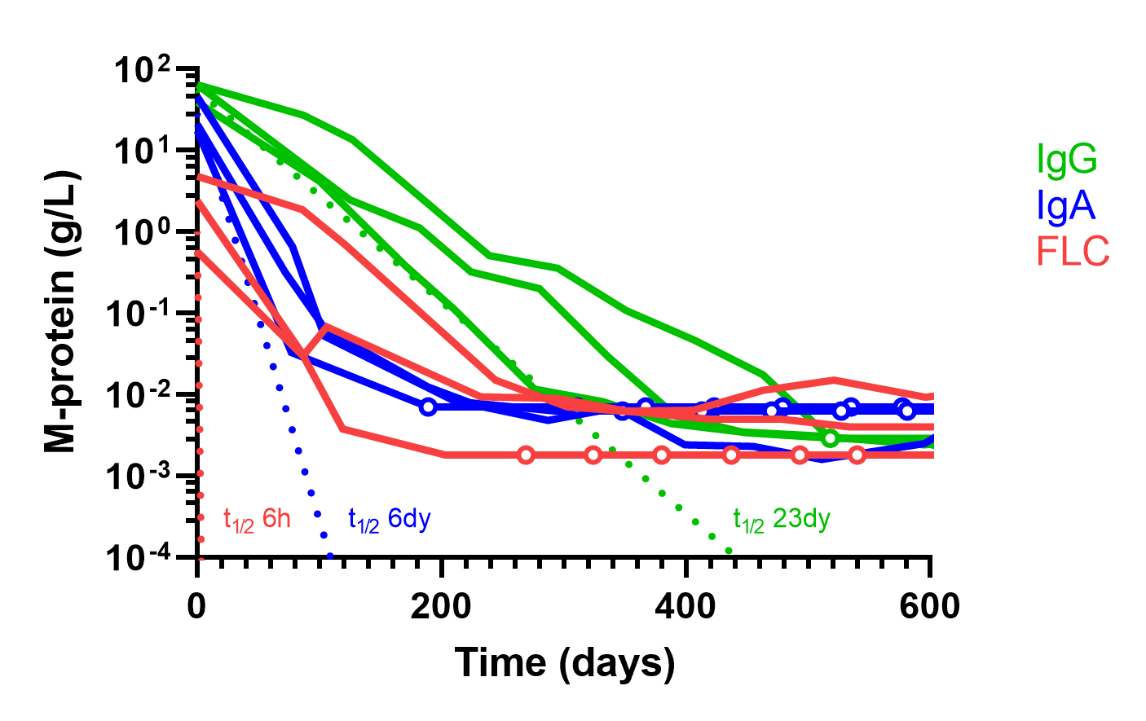
**

**Supplementary Figure 10: Distinct clearance rates for IgG class, IgA class, and free light chain M-proteins.** Dynamic MS-MRD data is shown for 3 patients with an IgG class M-protein (green), three with an IgA class M-protein (blue), and all patients with FLC M-protein (red). Samples negative by MS-MRD are shown with open symbols. Also shown is the theoretical clearance of IgG, IgA, and FLC based on half-lives reported in literature. There is a clear difference between patients with IgA M-protein who have a faster clearance than the patients with IgG M-protein, reflecting the expected difference in half-life between IgG (23 days) and IgA (6 days) isotypes. The dataset also includes 3 patients with a FLC M-protein. While the reported half-life for FLC is 6 hours, the rate of decrease was similar to IgA and IgG M-proteins. The IgA M-protein concentration reaches a plateau at approximately 200 days and IgG M-protein at approximately 400 days, which may indicate that an equilibrium is reached between clearance and remaining M-protein production in the patient. Therefore, in assessing the response to treatment the M-protein isotype and the associated clearance parameters should be taken into account.


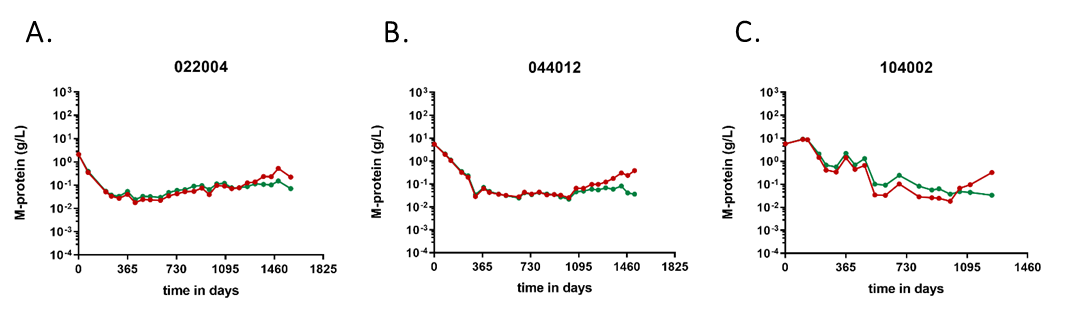


**Supplementary Figure 11: Patients suspected of free light chain escape.** For 3 patients there was a divergence between the heavy chain (green) and light chain (red) peptide data of the M-protein. In all 3 patients, the light chain peptide showed a distinct increase compared the heavy chain peptide which could indicate a light chain escape, where the production of FLC starts to exceed the production of intact M-protein at the time of progression.

**
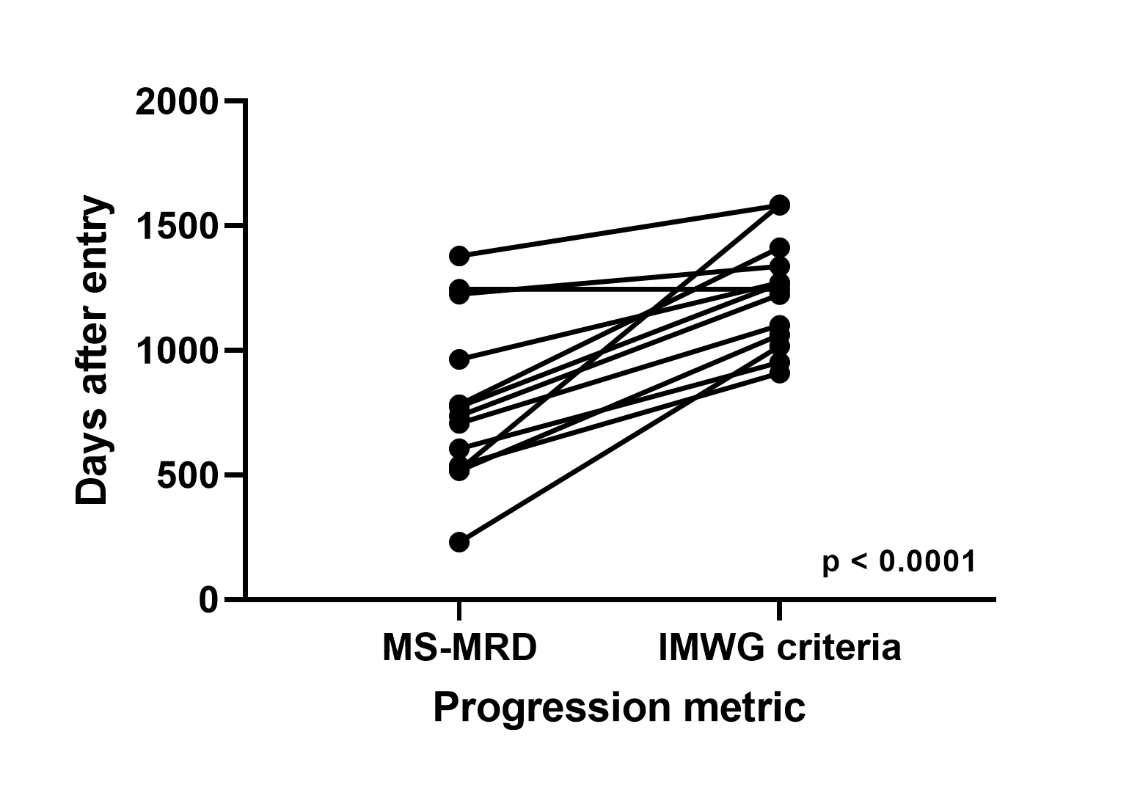
**

**Supplementary Figure 12. Earlier detection of progression by MS-MRD.** Shown are data from all 13 patients in the cohort who progressed from complete response during the serum collection period. The average time until disease progression was 1228 days. Each dot symbolizes the time elapsed until progression as assessed by either MS-MRD or by the original data available from the IFM 2009 trial which used the IMGW criteria; for each patient the data from MS-MRD and IFM 2009 trial are connected by a line. On average, MS-MRD detected progression significantly (p< 0.0001 in a paired t-test) 442 days earlier than in the trial.

**Supplementary Tables**

**Supplementary Table 1: Overview patient information**

| Patient ID | Treatment Arm | PFS in Months | M-protein Isotype | Follow Up in Days | Total Sera |
| --- | --- | --- | --- | --- | --- |
| 001002 | A | 40 | IgA-lambda | 1268 | 21 |
| 001004 | B | 40 | IgG-kappa | 1238 | 23 |
| 007003 | B | 51 | IgG-kappa | 1429 | 24 |
| 015001 | A | 62 | IgG-kappa | 1888 | 25 |
| 015002 | A | 42 | IgG-lambda | 1273 | 18 |
| 015005 | A | 27 | IgG-kappa | 894 | 13 |
| 017007 | B | 37 | IgA-lambda | 1017 | 18 |
| 019004 | B | 36 | IgG-lambda | 1092 | 19 |
| 019005 | B | 69 | IgG-lambda | 1758 | 29 |
| 019007 | B | 89 | IgG-kappa | 1693 | 27 |
| 020001 | B | 28 | IgA-kappa | 909 | 18 |
| 020004 | B | 51 | IgG-kappa | 1491 | 27 |
| 022004 | B | 49 | IgA-lambda | 1490 | 25 |
| 022006 | A | 88 | IgG-kappa | 1738 | 29 |
| 024004 | A | 32 | IgG-lambda | 1063 | 19 |
| 025002 | B | 79 | IgG-kappa | 1698 | 26 |
| 025007 | A | 95+ | IgG-kappa | 1481 | 24 |
| 034005 | B | 44 | IgG-kappa | 1412 | 23 |
| 034008 | A | 29 | IgA-kappa | 952 | 16 |
| 034017 | B | 91 | FLC-kappa | 1576 | 26 |
| 036001 | B | 34 | IgG-lambda | 1100 | 17 |
| 038008 | A | 40 | IgG-kappa | 1218 | 16 |
| 042006 | A | 59 | IgA-lambda | 1538 | 24 |
| 044012 | B | 55 | IgG-kappa | 1519 | 27 |
| 044018 | B | 30 | FLC-kappa | 1010 | 17 |
| 047001 | A | 89 | IgA-kappa | 1438 | 21 |
| 052003 | B | 38 | IgA-kappa | 1224 | 22 |
| 052005 | A | 49 | FLC-lambda | 1584 | 26 |
| 052007 | B | 49 | IgG-kappa | 1567 | 27 |
| 054002 | A | 58 | IgG-kappa | 1681 | 25 |
| 054006 | A | 27 | IgG-kappa | 916 | 13 |
| 067005 | B | 95+ | IgA-kappa | 1526 | 27 |
| 091004 | B | 84 | IgG-kappa | 1615 | 26 |
| 091006 | A | 86+ | IgG-kappa | 1512 | 24 |
| 091010 | A | 92+ | IgG-lambda | 1418 | 23 |
| 092002 | B | 98+ | IgA-lambda | 1098 | 17 |
| 099006 | B | 77 | IgG-kappa | 1498 | 23 |
| 104002 | B | 41 | IgG-kappa | 1245 | 19 |
| 152002 | A | 96 | IgG-lambda | 1838 | 31 |
| 152005 | B | 105+ | IgG-kappa | 1806 | 30 |
| 152018 | B | 90 | IgG-kappa | 1407 | 23 |

FLC; free light chain

**Supplementary Table 2: Overview of clonotypic peptide masses, charge states, and optimized collision energy**

| Patient ID | Heavy chain *m/z* (Charge state) | | | NCE | Light chain *m/z* (Charge state) | | | NCE |
| --- | --- | --- | --- | --- | --- | --- | --- | --- |
|  | **Sequence** | **M-protein** | **SIL standard** |  | **Sequence** | **M-protein** | **SIL standard** |  |
| 001002 | YWGQGTLVTVSSASPTSPK | 983.4995 (2+) | 987.5066 (2+) | 21 | VEDGDEADYYC[+57]QVWDSSSAHVVFGGGTR | 1036.1088 (3+) | 1039.4449 (3+) | 30 |
| 001004 | SAGYYWSWIR | 644.8091 (2+) | 649.8132 (2+) | 20 | LEPEDFAVYFC[+57]QHYNTSPLFTFGPGTK | 1055.8269 (3+) | 1058.4983 (3+) | 28 |
| 007003 | AGTFDYWGQGTLVTVSSASTK | 726.0234 (3+) | 728.6948 (3+) | 22 | SSHSLYSSHNK | 623.7942 (2+) | 627.8013 (2+) | 24 |
| 015001 | GLEWVTVIWHDGSK | 542.9473 (3+) | 545.6187 (3+) | 18 | WLAWYQQKPGK | 468.9189 (3+) | 471.5903 (3+) | 26 |
| 015002 | GLEWVASISSK | 588.8166 (2+) | 592.8237 (2+) | 23 | SEDEAEYYC[+57]ASWDVSR | 983.8916 (2+) | 988.8958 (2+) | 18 |
| 015005 | GSGYTFIDYYIHWVK | 616.9700 (3+) | 619.6414 (3+) | 22 | LEPEDFAVYFC[+57]QQYGSSPR | 765.0125 (3+) | 768.3486 (3+) | 23 |
| 017007 | GLEWVSLITWDAGSTYYADSVK | 821.0689 (3+) | 823.7403 (3+) | 18 | FSGSNSGSMATLTISR | 808.3909 (2+) | 813.3950 (2+) | 27 |
| 019004 | ALEWLAHIFSNDEK | 558.2825 (3+) | 560.9539 (3+) | 19 | QSVLTQPPSVSAAPGQR | 861.9603 (2+) | 866.9644 (2+) | 23 |
| 019005 | GLEWIGEIYHNGNANYNPTLK | 801.7291 (3+) | 804.4005 (3+) | 18 | LMIYEVSGRPSGVSNR | 588.9736 (3+) | 592.3097 (3+) | 18 |
| 019007 | SDGGTTDYSAPVK | 649.2990 (2+) | 653.3061 (2+) | 21 | SSQSLLHSNGNNYLDWYLQKPGQSPQLLIYLGSNR | 998.5079 (4+) | 1001.0099 (4+) | 18 |
| 020001 | EVQLVESGGALVEPGGSLR | 949.0049 (2+) | 954.0090 (2+) | 18 | LLIFAASNLQSGVPSR | 836.9727 (2+) | 841.9768 (2+) | 20 |
| 020004 | ASGGIFTNSIITWVR | 811.4385 (2+) | 816.4426 (2+) | 27 | ASSLEGGVPSR | 530.2751 (2+) | 535.2792 (2+) | 23 |
| 022004 | GLEWIGYIYHSGSTLYNPSLK | 800.0741 (2+) | 802.7455 (3+) | 27 | DNTANQLVFGGGTK | 711.3546 (2+) | 715.3617 (2+) | 21 |
| 022006 | YNWFDEWGQGTLVIVSSASTK | 796.7217 (2+) | 799.3931 (3+) | 27 | LLIHDASR | 462.7667 (2+) | 467.7708 (2+) | 27 |
| 024004 | GLEWVSSIGTVAADVYFPVSVR | 588.8113 (4+) | 591.3134 (4+) | 28 | VEAGDEADYYC[+57]QVWDTTTNQGVFGGGTK | 1023.4453 (3+) | 1026.1167 (3+) | 18 |
| 025002 | GLEWVSYISSSGTYTNYADSVK | 809.7165 (3+) | 812.3879 (3+) | 21 | LLIYDTSNLETGVPSR | 889.4702 (2+) | 894.4743 (2+) | 18 |
| 025007 | QAPGQGLEWMGAHIPIFGTPNYAQNFQGR | 1062.5173 (3+) | 1065.8534 (3+) | 27 | ASQSVSGSYFAWYQQKPGQAPR | 815.0645 (3+) | 818.4006 (3+) | 30 |
| 034005 | NEFSLNLR | 496.7616 (2+) | 501.7658 (2+) | 19 | APQLLIYAASNLK | 701.4087 (2+) | 705.4158 (2+) | 21 |
| 034008 | SLPIGELTSWGPGTLVTVSSASPTSPK | 890.4726 (3+) | 893.1440 (3+) | 21 | EIELTQSPGTLSLSPGEGATLSC[+57]R | 835.0830 (3+) | 838.4191 (3+) | 23 |
| 034017 | - | FLC | - | - | LLIYTASSLQNGVPSR | 859.9754 (2+) | 864.9796 (2+) | 18 |
| 036001 | GSSLSYYGMDVWGQGTTVTVSSASTK | 890.4182 (3+) | 893.0896 (3+) | 22 | QKPGQSPVLLIYQDNK | 610.0036 (3+) | 612.6750 (3+) | 18 |
| 038008 | DGGSSSTWYR | 372.4966 (3+) | 375.8327 (3+) | 20 | AIQLTQSPSSLSASVEDR | 944.9842 (2+) | 949.9883 (2+) | 25 |
| 042006 | EGQLVESGGALVEPGGSLR | 927.9814 (2+) | 932.9856 (2+) | 21 | LLTQPPSASGTPGLR | 747.9174 (2+) | 752.9215 (2+) | 24 |
| 044012 | SQGGLIPLDSWGQGTLVAVSSASTK | 820.4307 (3+) | 823.1021 (3+) | 18 | LLIYDASTLEDGVPSR | 874.9569 (2+) | 879.9610 (2+) | 18 |
| 044018 | - | FLC | - | - | LLIHATSNLQSGVPSR | 564.9846 (3+) | 568.3207 (3+) | 20 |
| 047001 | GLEFIGTMYYTGSTYYNPSLK | 802.3838 (3+) | 805.0552 (3+) | 19 | LEPEDFAMYYC[+57]QQYGDSLWTFGQGTK | 1045.4564 (3+) | 1048.1278 (3+) | 23 |
| 052003 | GLEWIGYVYYSGSTSYNPSLK | 795.3864 (3+) | 798.0578 (3+) | 21 | APQLLIHTASNLQSGVPSR | 663.7007 (3+) | 667.0368 (3+) | 21 |
| 052005 | - | FLC | - | - | VTISC[+57]SGSSSNIGR | 712.8435 (2+) | 717.8477 (2+) | 22 |
| 052007 | DDSTNTVFLQMNNVR | 877.4123 (2+) | 882.4165 (2+) | 22 | STLQGGVPSR | 501.2724 (2+) | 506.2765 (2+) | 23 |
| 054002 | GLEWMGVIYPGDSDSR | 891.4118 (2+) | 896.4159 (2+) | 18 | ASQNINFWLAWYQQKPGK | 727.0410 (3+) | 729.7124 (3+) | 25 |
| 054006 | QAPGTGLEWIAYISTGSTAIHYADSVK | 946.1433 (3+) | 948.8147 (3+) | 27 | SVSSDLAWYQQK | 706.3462 (2+) | 710.3533 (2+) | 21 |
| 067005 | GMC[+57]VSWIR | 1045.4564 (3+) | 509.7455 (2+) | 21 | APILLIYDASNLETGVPSR | 677.0370 (3+) | 680.3731 (3+) | 20 |
| 091004 | GLEWVSYISSSGSTIYHAASVQGR | 852.4223 (3+) | 855.7584 (3+) | 27 | VTITC[+57]QASQDISNYLNWYQQK | 854.0761 (3+) | 856.7475 (3+) | 27 |
| 091006 | DVDYHGMDVWGQGTTVTVSSASTK | 847.7236 (3+) | 850.3950 (3+) | 18 | SQGVTTWMAWYR | 743.3508 (2+) | 748.3549 (2+) | 21 |
| 091010 | LSC[+57]AAPGFTLNSYAMHWVR | 727.6838 (3+) | 731.0199 (3+) | 30 | YVSWFQQHPGK | 459.5630 (3+) | 462.2344 (3+) | 22 |
| 092002 | TVSGFSLSNPR | 582.8040 (2+) | 587.8082 (2+) | 21 | SVSGSPGQSVTISC[+57]TGTNSDVGGYDYVSWYQQHPGK | 1269.2393 (3+) | 1271.9107 (3+) | 27 |
| 099006 | GSGYTFSENAIHWVR | 575.2777 (3+) | 578.6138 (3+) | 28 | ASEGVSSYYLAWYQQK | 940.4467 (2+) | 944.4538 (2+) | 22 |
| 104002 | LSC[+57]AASGFTFSNYGMHWVR | 730.9998 (3+) | 734.3359 (3+) | 27 | TNWLWTFGQGTK | 719.8593 (2+) | 723.8664 (2+) | 22 |
| 152002 | NQVVLTMTNMDPMDTATYYC[+57]AHR | 911.4027 (3+) | 914.7388 (3+) | 26 | QPVLTQPPSSSASPGESAR | 948.4765 (2+) | 953.4806 (2+) | 27 |
| 152005 | GLEWLAVISGDETTK | 809.9198 (2+) | 813.9269 (2+) | 20 | LLIYAASTLENGVPSR | 852.4700 (2+) | 857.4741 (2+) | 18 |
| 152018 | LDHGGNPFMYWGPGTLVAVSSASTK | 864.7566 (3+) | 867.4280 (3+) | 23 | FSGSGSVTDFTLEISR | 851.9178 (2+) | 856.9219 (2+) | 27 |

FLC; free light chain, NCE; normalized collision energy, SIL; stable isotope labelled

**Supplementary Table 3: Method comparison NGS-MRD with MS-MRD results**

|  | NGS-MRD positive | NGS-MRD negative |
| --- | --- | --- |
| MS-MRD positive | 63 | 14 |
| MS-MRD negative | 3 | 1 |
